# Improved Direct Current Electrical Properties of Crosslinked Polyethylene Modified with the Polar Group Compound

**DOI:** 10.3390/polym11101624

**Published:** 2019-10-08

**Authors:** Chengcheng Zhang, Jianxin Chang, Hongyu Zhang, Chunyang Li, Hong Zhao

**Affiliations:** Key Laboratory of Engineering Dielectrics and Its Application, Ministry of Education, Harbin University of Science and Technology, Harbin 150080, China; 17645760291@163.com (J.C.); zhy925027323@126.com (H.Z.); lichunyang_hust@163.com (C.L.)

**Keywords:** space charge, trap, conduction, breakdown

## Abstract

To suppress space charge accumulation and improve direct current (DC) electrical properties of insulation materials, crosslinked polyethylene modified with 2-(4-benzoyl-3-hydroxyphenoxy) ethyl acrylate (XLPE/BHEA) containing polar functional groups was prepared by melt blending. The gel content, thermal elongation, tensile strength, elongation at break, elasticity modulus, differential scanning calorimetry (DSC) and X-ray photoelectron spectra (XPS) measurement results demonstrated that the BHEA could slightly enhance the crosslinking of polyethylene (PE) and affect the mechanical properties and crystallization of XLPE, and the BHEA molecule was not easy to precipitate from XLPE after the crosslinking process. XLPE modified with 3.0 phr (parts per hundreds by weight) BHEA could effectively suppress space charge accumulation, reduce DC conduction and improve DC breakdown strength of XLPE at a higher temperature. Deeper traps were introduced in XLPE/BHEA composites due to the polar functional groups in BHEA, which could raise the potential charge injection barrier and reduce the charge carrier number and mobility to suppress space charge accumulation and reduce the conduction current density.

## 1. Introduction

The formation and accumulation of space charges has become one of the major problems that urgently needs to be solved for the development of high voltage direct current (HVDC) cable polyethylene (PE) insulation materials. The space charges are formed by the transfer and storage of the charge carriers injection by the electrodes and the ionization of the impurities or additives such as antioxidants, etc., in the bulk of PE insulation materials, which could lead to local electric field distortion and the local field may be eight times more than the applied electric field especially when the polarity of the line is inverted [1,2,3,4]. The distortion of the electric field caused by space charge accumulation could affect the conduction, breakdown and ageing phenomena of PE insulation materials, and reduce the long-term reliability and service life of direct current (DC) cables [5].

To suppress space charge accumulation and improve DC electrical properties of PE insulation materials, both super-clean materials with less additives and impurities and modified PE have been extensively investigated to reduce the origin of ion charges and inhibit the formation of electron charges. The super-clean materials have a low crosslinking extent and poor oxidation resistance, and are unable to improve the insulation quality further due to high costs and technology involved in the purification process [6]. PE is often modified by incorporating organic or inorganic additives and nanofillers, blending them with other polymers and grafting, etc. Nanofillers such as MgO, SiO_2_, Al_2_O_3_, ZnO, BaSrTiO_3_ nanoparticles or graphene oxide can suppress space charge accumulation in PE, and the content, size, surface modification of nanofillers and ambient conditions are known to have an impact on space charge distribution of modified PE [7,8,9,10,11,12,13]. The introduction of deep traps was considered to be the mechanism of suppressing space charge accumulation in the nanocomposites [14,15]. However, the uniform dispersion of nanofillers with a small size is difficult to achieve, and the properties of nanocomposites are susceptible to the dispersion and ambient conditions and could be severely deteriorated, resulting in space charge accumulation again [16]. It was reported that polar groups such as carbonyl functional groups and conjugated double bonds would create deep traps that captured electric charge carriers in polymer materials in the framework of Density Functional Theory (DFT) [17,18]. Kwang S. Suh et al. modified low density polyethylene (LDPE) by grafting acrylic acid and maleic anhydride containing polar groups using dicumyl peroxide (DCP) as an initiator and found that the heterocharges in LDPE decreased with the increase of acrylic acid and maleic anhydride graft ratio due to an enhanced charge trapping at carbonyl groups or ring structure [19,20]. However, air bubble defects easily formed in the insulation materials during the preparation process due to the low boiling point of acrylic acid and maleic anhydride and affected the performance of insulation materials. The crosslinked polyethylene (XLPE) modified by the voltage stabilizer with a high deep trap level demonstrated an excellent ability on space charge suppression at different temperatures and the effect of voltage stabilizers on the space charge inhibition may have been related to the types of functional groups it contained [21]. XLPE with the network structure has better thermal and chemical stability, and insulation and mechanical performance. Crosslinking is generally achieved through the use of peroxides, silanes or high energy radiation. The chemical method by peroxides is one of the most usual methods. Crosslinking PE with organic peroxides gives rise to stronger polymers with better resistance to stress cracking [22,23].

2-(4-benzoyl-3-hydroxyphenoxy) ethyl acrylate (BHEA) is a type of compound with many kinds of polar functional groups and a high boiling point, which may introduce deep traps in PE and eliminate air bubble defects formed during the preparation process. In this paper, to suppress space charge accumulation and further improve direct current electrical properties of insulation materials, XLPE modified with BHEA was prepared by melt blending. The migration of BHEA and the interference with the crosslinking, crystallization and mechanical properties were investigated by X-ray photoelectron spectra, the Soxhlet extraction method, thermal elongation, differential scanning calorimetry (DSC), tensile strength, elongation at break and elasticity modulus. The influence of polar functional groups on the space charge injection and accumulation was discussed. Furthermore, the conduction and breakdown performance with temperature was also studied.

## 2. Materials and Methods

### 2.1. Materials

**Figure 1:**
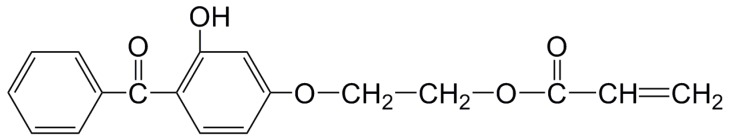
Chemical structural formula of 2-(4-benzoyl-3-hydroxyphenoxy) ethyl acrylate.

Low density polyethylene (LDPE, LD200GH) pellets with a density of 0.922 g/cm^3^ and a Melt Flow Index (MFI) of 2.0 g/10 min were supplied by Sinopec Beijing Yanshan Company, Beijing, China. 2-(4-benzoyl-3-hydroxyphenoxy) ethyl acrylate (BHEA) was purchased from J&K Scientific Ltd., Beijing, China and the structure is shown in Figure 1. Dicumyl peroxide (DCP) was obtained from Sinopec Shanghai Gaoqiao Petrochemical Co. Ltd., Shanghai, China. All of the aforementioned chemicals were used as received without further purification.

### 2.2. Preparation of Crosslinked Polyethylene (XLPE) Modified with 2-(4-Benzoyl-3-Hydroxyphenoxy) Ethyl Acrylate 

XLPE modified with BHEA (XLPE/BHEA) was prepared by melt blending. LDPE pellets were put into the mixer at 110 °C until they were completely molten. A certain amount of BHEA was added into the mixer and blended with LDPE for about 5 min. Then 2.0 phr (parts per hundreds by weight) DCP was added in for the appropriate crosslinking extent [24] and blended for only about 3 min to prevent DCP decomposition and a pre-crosslinked reaction. The obtained mixture was hot compression-moulded using a flat vulcanizing machine at 110 °C under a pressure of about 15 MPa, and then crosslinked under the same pressure at 175 °C for 30 min. Finally, the film specimens were removed and cooled to room temperature. XLPE, XLPE/BHEA-0.3, XLPE/BHEA-1.0 and XLPE/BHEA-3.0 were obtained and indicated XLPE modified with 0, 0.3, 1.0 and 3.0 phr BHEA, respectively. XLPE/BHEA composites were heated in a vacuum oven at 80 °C for 24 h to remove the residual crosslinking byproducts and moisture for different tests. Similarly, the crosslinkable PE specimen modified with 3.0 phr BHEA was prepared under the process as described above only without the crosslinking treatment at 175 °C for 30 min.

### 2.3. Characterization

Analysis of X-ray photoelectron spectra (XPS) was performed on a Thermo Fisher ESCALAB 250Xi electron spectrometer (USA) with an Al Kα X-ray source (Thermo Fisher Scientific, Waltham, MA, USA) and a pass energy of 20 eV. The survey spectra and the high resolution C1s and O1s spectra of crosslinkable PE modified with 3.0 phr BHEA and XLPE modified with 3.0 phr BHEA were obtained. Using a XPS peak fitting program (XPSPEAK Version 4.0, The Chinese University of Hong Kong, Hong Kong, China), the C1s and O1s peaks for the specimens before and after crosslinking were deconvoluted into several subpeaks of functional groups.

The gel content and thermal elongation are usually adopted to characterize the extent of XLPE crosslinking. The gel content was assessed by the Soxhlet extraction method. Higher gel content is an indication of more crosslinking. The crosslinked specimen of *M*_1_ with a precision of 0.001 g was cut into 1 × 1 cm^2^ pieces and refluxed in boiling xylene solution for about 12 h. The non-crosslinked fraction in XLPE was supposed to dissolve completely in the xylene and the remaining insoluble fraction was considered to be crosslinked elastomer matrix. The insoluble residue was dried at 150 °C in a vacuum oven to a constant weight and then weighed accurately as *M*_2_. The gel content, from duplicates runs, was calculated using the following Equation (1) [25]:

(1) $$\mathrm{Gel}\;\mathrm{Content}(\%)=M_{2}/M_{1}\times 100$$

In the thermal elongation experiment, dumb-bell shaped crosslinked specimen was strained with a stress of about 0.2 MPa in an oven at 200 °C. After equilibration for 10 min, based on analysis of triplicate samples, the thermal elongation was given by the following Equation (2) [26]:

(2) $$\mathrm{Thermal}\;\mathrm{Elongation}(\%)=L/L_{0}\times 100$$

where *L*_0_ is the gauge length, *L* is the elongation length under stress. Lower thermal elongation is an indication of more crosslinking.

The effect of BHEA on the crystallization process of XLPE was analyzed by differential scanning calorimetry (DSC, Mettler Toledo DSC 1, Columbus, OH, USA) after eliminating the thermal history with the heating velocity of 10 °C/min under a nitrogen atmosphere at the measurement temperature range of 25–150 °C. Based on the DSC melting thermogram of the second heating as shown in Figure S1 (Supplementary Materials), the degree of crystallinity of pristine XLPE and XLPE/BHEA composites was calculated using the following Equation (3) [24,27]:

(3) $$\mathrm{Degree}\;\mathrm{of}\;\mathrm{Crystallinity}(\%)=\Delta H_{m}/\Delta H_{100}\times 100$$

where Δ*H_m_* is the enthalpy of fusion normalized by weight, Δ*H_100_* is the enthalpy of fusion for 100% crystalline PE which was taken as 293.6 J/g.

### 2.4. Mechanical Properties Measurement

The mechanical properties of the specimen were measured by using an electronic universal testing machine (Model CMT6000, MTS Industrial Systems Co., Ltd., Shenzhen, China) according to the Standardization Administration of China (SAC) Publication No. GB/T 1040.1-2006 and GB/T 1040.2-2006 (Plastics-Determination of tensile properties) at a tensile speed of 50 mm/min. The stress–strain curves of pristine XLPE and XLPE modified with BHEA are shown in Figure S2 (Supplementary Materials). The tensile strength, elongation at break and elasticity modulus of each XLPE material was finally averaged after five repeated measurements to decrease the experiment inaccuracy.

### 2.5. Electrical Performance Measurement

The space charge measurement of the specimen was performed with a pulse electro-acoustic (PEA) system. Silicone oil was used as acoustic coupling to ensure good acoustic transmission between the specimen and the electrode. A semi-conductive layer made of carbon black loaded PE was attached to the upper electrode. Calibration was conducted at a DC electric field of 3 kV/mm for 5 min to minimize the influence on space charge accumulation. In the experiment, a 40 kV/mm DC electric field was applied to the specimen with an average thickness of 300 μm for 40 min at room temperature to reach a steady state or quasi steady state and then removed. The measurement signals both in the polarization and depolarization process were collected over time by a digital oscilloscope, and the data were processed using a calibration trace and a deconvolution technique to restore the original signal.

The three-electrode system was adopted to measure DC electrical conduction current to achieve a relatively uniform electric field under the measurement electrodes. The test system and the specimen with an average thickness of 200 μm and aluminum foil electrodes on both sides were placed in an oven, which could eliminate the interference of external signals and control the measurement temperature from 30 to 70 °C. A EST122 picoammeter was used to collect the quasi stationary current under different DC electric fields between 1 to 50 kV/mm at different measurement temperatures with a precision of up to 10^−14^ A.

DC breakdown test was conducted by placing the specimen with an average thickness of about 50 μm between two opposing cylindrical electrodes with a diameter of 25 mm immersed in silicone oil to isolate them from the air, prevent surface flashover and control the temperature in the range of 30–70 °C in the measurements. The applied DC voltage was increased at a rate of 600 V/s until the breakdown occurred. The resulting breakdown data were statistically treated by using two-parameter Weibull statistical distribution method after 30 measurements to decrease the experiment inaccuracy [28].

The depolarization current was tested by a thermally stimulated current (TSC) method under a linear temperature program. The specimen with a thickness of about 100 μm was polarized with a DC electric field of 30 kV/mm for 30 min under vacuum condition at 60 °C and then rapidly cooled down to −50 °C under the application of the polarization voltage. Following this, the electric field was removed and the external circuit was shorted until the discharge current was less than 5 pA. Finally, the depolarization current was measured continuously with the temperature raised up to 170 °C at a heating rate of 3 °C/min.

## 3. Results and Discussion

**Figure 2:**
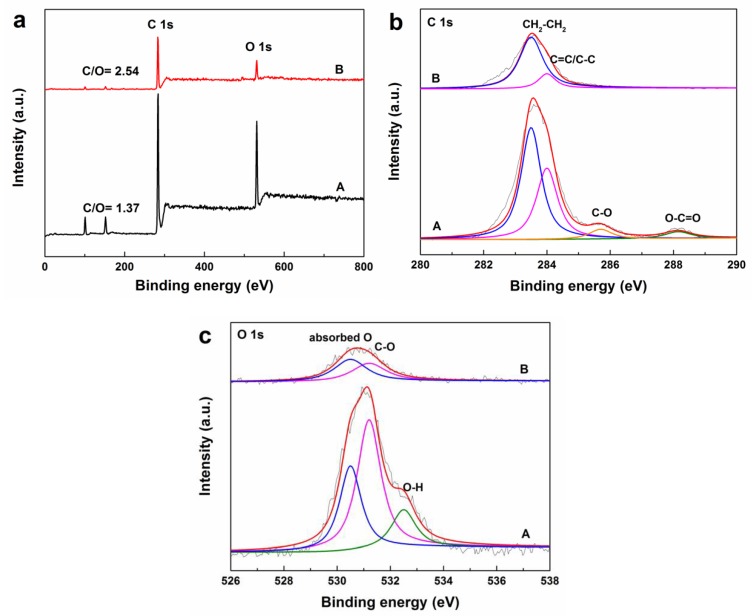
XPS analysis of crosslinkable PE modified with 3.0 phr BHEA (**A**), XLPE modified with 3.0 phr BHEA (**B**), (**a**) survey, (**b**) C1s and (**c**) O1s.

BHEA has poor compatibility with PE. It will migrate easily to a cable insulation surface and be removed from the surface in a short time. To investigate the effect of crosslinking on the migration of BHEA, the chemical composition and structure of crosslinkable PE modified with 3.0 phr BHEA and XLPE modified with 3.0 phr BHEA were analyzed using XPS as shown in Figure 2. The characteristic signals for C and O were clearly detected at around 283.6 and 531.3 eV, respectively in XPS survey spectra (Figure 2a). The C/O atomic ratios measured were calculated to be 1.37 and 2.54 for the specimen surface of crosslinkable PE modified with BHEA and XLPE modified with BHEA, respectively. For crosslinkable PE modified with BHEA, the high resolution C1s spectrum could be deconvoluted into four components, the bands at 283.5, 284.0, 285.7 and 288.2 eV were attributed to CH_2_–CH_2_, C=C/C–C, C–O, O–C=O, respectively [29,30,31,32]. The O1s spectrum could be deconvoluted into three components, with the binding energies at 530.5, 531.2 and 532.5 eV. The peak at 530.5 eV was related to the absorbed oxygen species, while the peaks at 531.2 and 532.5 eV were attributed to C–O and O–H, respectively [31,33,34]. After the crosslinking reaction, the relative peak intensity of C=C/C–C of C1s and C–O of O1s decreased, and the bands for C–O and O–C=O of C1s and O–H of O1s which may have been related to the BHEA molecular disappeared. The XPS spectra indicated that there were much less BHEA molecules extracted on the surface of XLPE/BHEA composites suffering from a higher temperature preparation process, and confirmed that the crosslinking reaction could reduce the migration and extraction of the BHEA molecule from XLPE/BHEA composite specimen.

The extent of XLPE crosslinking could significantly affect its mechanical and electrical properties, and stability. In this paper, gel content and thermal elongation were adopted to characterize the extent of XLPE crosslinking, and the relative results are shown in Table 1. With the BHEA content increasing, the gel content of XLPE/BHEA composites rose and the thermal elongation reduced slightly, which indicated that the addition of BHEA did not interfere with the crosslinking process of PE and the crosslinking extent of XLPE/BHEA composites increased slightly. As shown in Table 1, the tensile strength of XLPE/BHEA composites increased firstly and then decreased a little, the elongation at break decreased slightly and the elasticity modulus increased a little as the BHEA content increased. The mechanical properties of XLPE changed unobviously after modification with BHEA, which may have been due to a slight increase in the extent of XLPE crosslinking after the addition of BHEA.

The melting temperature and degree of crystallinity of XLPE before and after adding BHEA with polar functional groups was investigated by DSC and the results are also shown in Table 1. The melting temperature of XLPE rose slightly after BHEA was added in and the differences were considered to be insignificant. The degree of crystallinity of XLPE/BHEA composites was smaller than that of pristine XLPE and with the BHEA content increasing, the degree of crystallinity of XLPE/BHEA composites reduced slightly. The increased crosslinking after the addition of BHEA could destroy the ordered structure and limit the activity of the PE chain to reduce the degree of crystallinity of XLPE.

**Figure 3:**
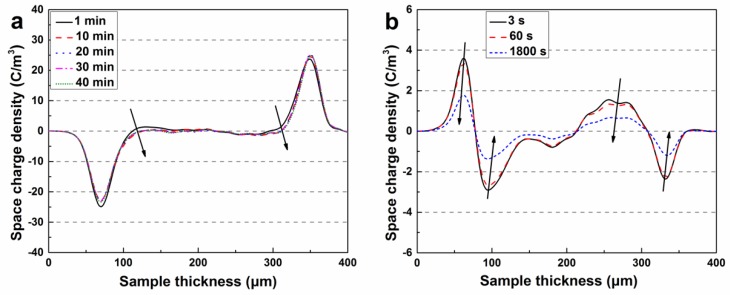
Space charge distributions in pristine XLPE during polarization (**a**) and depolarization (**b**).

**Figure 4:**
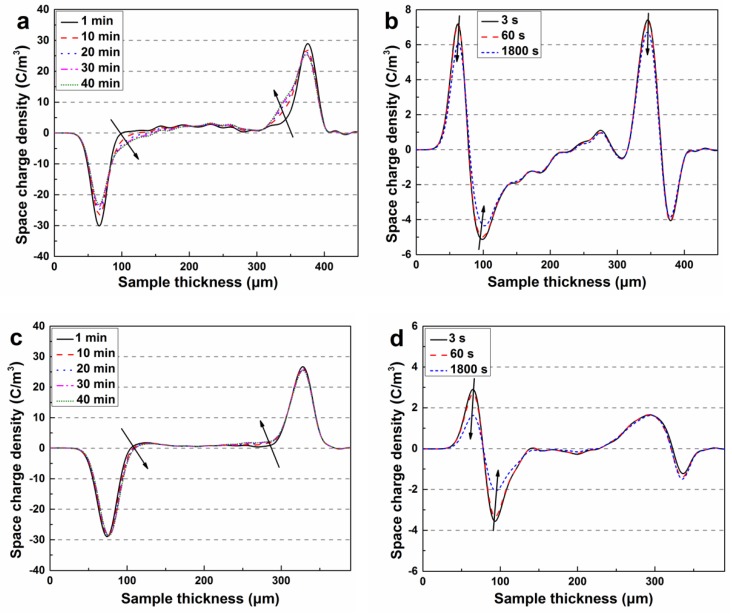

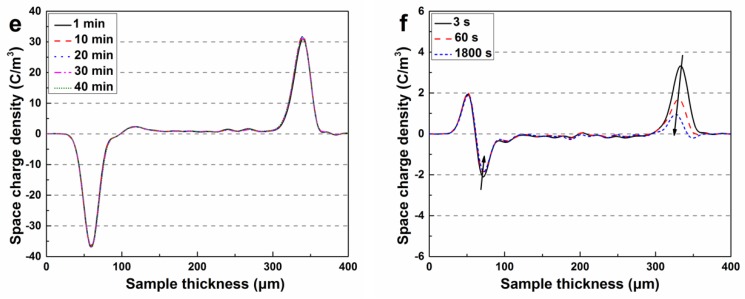
Space charge distributions in XLPE modified with BHEA during polarization and depolarization. (**a**,**b**) corresponding to XLPE/BHEA-0.3, (**c**,**d**) to XLPE/BHEA-1.0, (**e**,**f**) to XLPE/BHEA-3.0; (**a**,**c**,**e**) corresponding to polarization, (**b**,**d**,**f**) to depolarization.

Figure 3 and Figure 4 show the space charge behavior of pristine XLPE and XLPE modified with different BHEA contents subjected to a DC electric field of 40 kV/mm during polarization and depolarization, respectively. It can be seen from Figure 3 that the heterocharges accumulated in the vicinity of the anode in pristine XLPE, which may have been arising from the ionization of impurities from the crosslinking agent, catalyst and crosslinking by-products in XLPE, and the homocharges injected from the electrode appeared near the cathode soon after the electric field was applied and the amount of charges increased with the stressed time. At the same time, a large amount of positive and negative space charge packets were formed through the interior of pristine XLPE specimen. During the short-circuit process after 40 min of polarization, the space charges around the electrodes and the space charge packets in the interior of pristine XLPE decayed gradually.

As shown in Figure 4a,b, after adding 0.3 phr BHEA in XLPE, a large amount of homocharges were injected in and accumulated near the cathode and anode, and the injection depth increased with the polarization time, which resulted in the space charge packets slowly growing up and accumulating from the surface close to the electrodes to the interior of the material. However, it can be seen that from Figure 4a–f, with the BHEA content increasing, the amount of homocharges in the vicinity of the cathode and anode reduced, the injection depth of space charge packets was shallowed gradually, and the curve of charge density between cathode and anode tended to be smooth when XLPE was modified with 3.0 phr BHEA.

**Figure 5:**
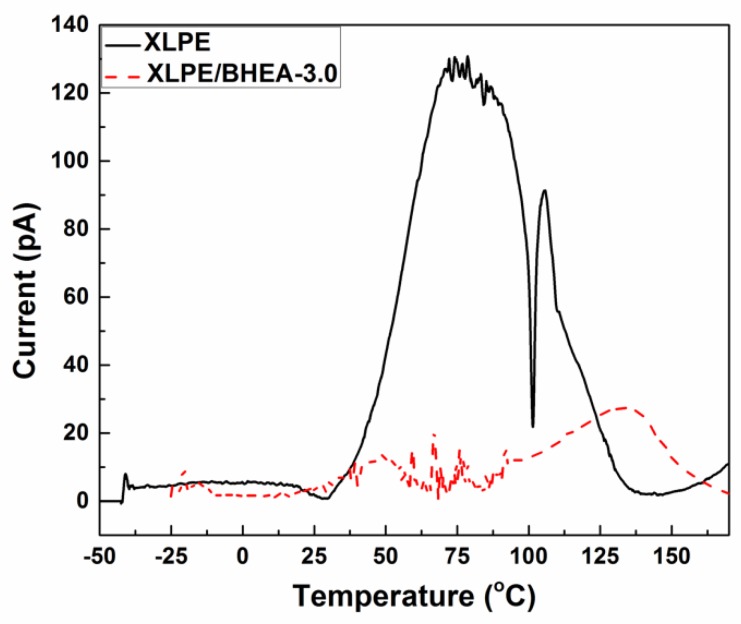
TSC curves of pristine XLPE and XLPE modified with 3.0 phr BHEA.

TSC spectra of pristine XLPE and XLPE modified with 3.0 phr BHEA are shown in Figure 5. The TSC current peak of pristine XLPE appeared at approximately 79 and 105 °C with the peak value of 128 and 91 pA, respectively. After modification with 3.0 phr BHEA, the value of the TSC current peak of XLPE at 79 and 105 °C reduced significantly and a new TSC current peak with the value of 27 pA in the high temperature region at about 133 °C emerged. XLPE is a semi-crystalline polymer material with the traps created by the physical defects in the amorphous region, the interface region between the crystalline and amorphous and crystalline region, and the chemical defects caused by chemical impurities [35,36]. For pristine XLPE, the cavity traps created by the physical defects mainly located in the interface regions between the crystalline and amorphous region will release charge and generate current at 79 °C during a linear temperature program process. When the temperature rose to 105 °C, the crystalline region melted and the charges captured in the traps induced by the crystal structure defects were detrapped and released. After BHEA was added in XLPE, polar functional groups containing carbonyl, hydroxyl, ether and ester group of BHEA would bring in deeper traps corresponding to the space charge relaxation temperature of 133 °C in the TSC curve. Under the low BHEA content, the deep traps were scarce and dispersive, which could play a role as charge trapping centers to trap electrons and holes [37]. Thus, under the low BHEA content, a large amount of homocharges accumulated near the cathode and anode. Under the high BHEA content, these deep traps increased and formed a dense trap layer near the cathode and anode, which could capture the homocharges injected from the electrodes when the DC electric field was applied, forming a space charge layer and an independent electric field in the vicinity of the electrodes. The effective electric field between the independent electric field and electrode reduced and the potential charge injection barrier was raised, which would further block the continuous charge injection from the electrodes and reduce the charge carrier number and mobility. Therefore, the release amount of charges in the shallower traps was significantly decreased as shown in the TSC curve of XLPE/BHEA composites in Figure 5. Meanwhile, these deep traps could capture the charge carriers to reduce the heterocharges from the ionization of impurities in the bulk of the material. Thus, the space charges in the interior of XLPE/BHEA composites with high BHEA content were significantly suppressed due to the deep traps introduced by the polar functional groups of BHEA [14,16].

**Figure 6:**
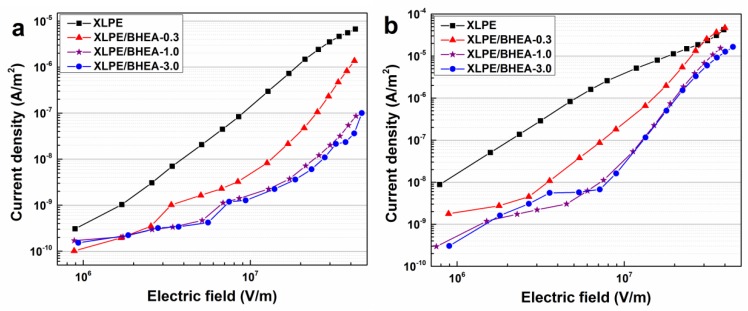

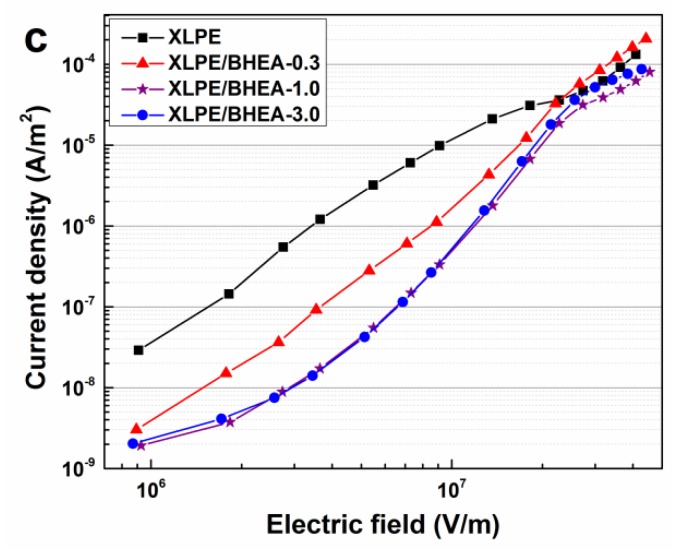
Dependence of DC conduction current density of pristine XLPE, XLPE/BHEA-0.3, XLPE/BHEA-1.0 and XLPE/BHEA-3.0 on the electric field at the measurement temperatures of 30 °C (**a**), 50 °C (**b**) and 70 °C (**c**).

Figure 6a–c show the dependence of DC conduction current density of pristine XLPE and XLPE modified with different BHEA contents on the electric field at 30, 50 and 70 °C. The conduction current density of pristine XLPE and XLPE/BHEA composites increased with the applied electric field and temperature increasing. At the measurement temperature of 30 °C, the conduction current density of XLPE/BHEA composites was significantly less than that of pristine XLPE at the whole applied electric field range, and first decreased significantly and then changed little with the BHEA content increasing. However, when the measurement temperature rose, under a lower applied electric field, the conduction current density of XLPE/BHEA composites was also always much less than that of pristine XLPE, nevertheless, under a higher applied electric field, the descent rate of the conduction current density of XLPE/BHEA composites was not remarkable and the conduction current density of XLPE modified with 0.3 phr BHEA slightly exceeded that of pristine XLPE when the applied electric field was over 30 and 23 kV/mm at the measurement temperature of 50 and 70 °C, respectively. 

The deep traps induced by the polar functional groups of BHEA in the XLPE/BHEA composites could capture and scatter the charge carriers in the transport process, and in addition, the space charge layer in the vicinity of the electrodes induced by the deep traps under the high BHEA content could suppress the further injection of charge carriers, which would reduce the charge carrier number and mobility in the amorphous regions and lead to a decrease in the conduction current density of XLPE/BHEA composites [38]. This was in accordance with the results of space charge measurements. The reason that the conduction current density of XLPE/BHEA composites increased relatively under a higher measurement temperature and applied electric field may have been due to the charge carriers captured in the shallow traps, which were activated and detrapped back to transport states, which made the conduction current density increase slightly, especially when the number of deep traps was small under the low BHEA content in XLPE. At different measurement temperatures, the dependence of the DC conduction current density of XLPE/BHEA composites on the electric field increased compared to that of pristine XLPE, which would reduce the maximum steady-state and the transient electric field, and make the electric field distribution more uniform in the cable insulation.

**Figure 7:**
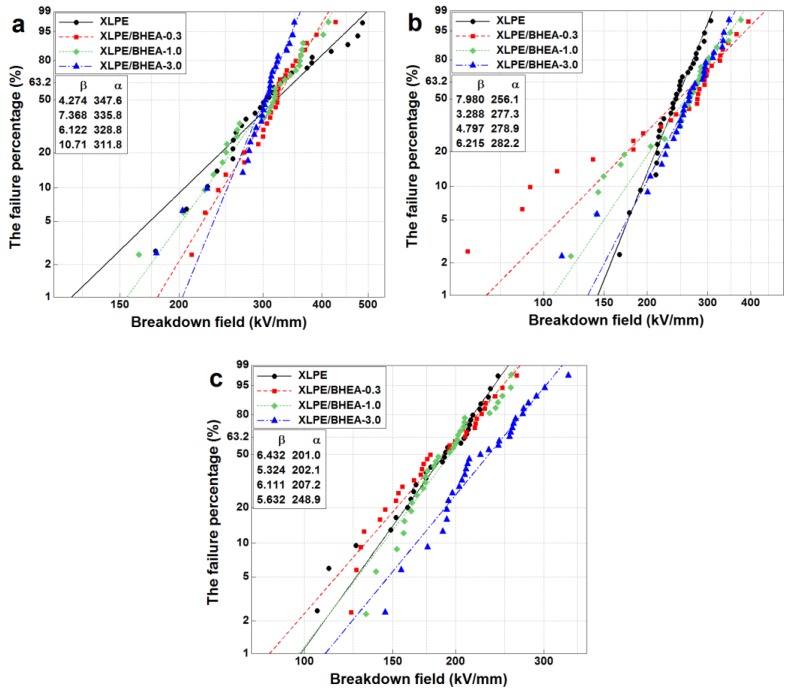
Weibull probability plots for DC breakdown strengths of pristine XLPE, XLPE/BHEA-0.3, XLPE/BHEA-1.0 and XLPE/BHEA-3.0 at the measurement temperatures of 30 °C (**a**), 50 °C (**b**) and 70 °C (**c**).

Figure 7 shows the two-parameter Weibull probability plots for DC breakdown strengths of pristine XLPE and XLPE modified with 0.3, 1.0 and 3.0 phr BHEA at different measurement temperatures with derived parameter values. In this, α is the scale parameter that represents the DC breakdown strength at the cumulative breakdown probability of 63.2% and is called the Weibull breakdown strength, while β the shape parameter that represents the inverse of data scatter. It was observed that at the measurement temperature of 30 °C, the Weibull DC breakdown strength of pristine XLPE was as high as 347.6 kV/mm. After adding 0.3 phr BHEA in XLPE, the DC breakdown strength reduced slightly to 335.8 kV/mm, and reduced further to 328.8 and 311.8 kV/mm as the BHEA content increased from 1.0 to 3.0 phr, close to that of pristine XLPE with a small drop. However, when the measurement temperature rose, the DC breakdown strength of XLPE/BHEA composites excelled that of pristine XLPE instead, although the increment of the breakdown strength was not remarkable when the BHEA content increased.

At a lower measurement temperature, the BHEA with highly polar functional groups could affect crystallization of XLPE, leading to a loose molecular arrangement and the free volume enlarging in some locations, which made the DC breakdown strength of XLPE/BHEA composites reduce slightly compared to that of pristine XLPE. At a higher measurement temperature, the breakdown strength of pristine XLPE and XLPE/BHEA composites all reduced due to the increase of the free electron content injected from the electrode, more energy obtained by the electrons and a softening phenomenon of the molecular chain [14]. When the measurement temperature rose, the motion of XLPE macromolecular chain strengthened and the XLPE chain was in a stretched conformation, which led to the aggregation structure and free volume of pristine XLPE and XLPE/BHEA composites inclining to be the same. Meanwhile, the deep traps induced by the polar functional groups of BHEA could capture and scatter the free charges, resulting in the reduction of the amount of free charge carriers and energy loss of the free charges, and the softening phenomenon could be weakened with the high crosslinking extent of XLPE/BHEA composites, which would improve the DC breakdown strength of XLPE/BHEA composites at a higher measurement temperature. The addition of BHEA in XLPE could improve the heat-resisting properties of XLPE insulation materials.

## 4. Conclusions

XLPE modified with BHEA containing polar functional groups was prepared by melt blending. The addition of BHEA would not interfere with and instead improve the crosslinking process of PE slightly and affect the mechanical properties and crystallization of XLPE slightly. The addition of 3.0 phr BHEA in XLPE could effectively suppress space charge accumulation and reduce DC electrical conduction, while change DC breakdown strength very little at a lower measurement temperature and enhance DC breakdown strength at a higher measurement temperature, which indicated that the addition of BHEA in XLPE could improve the heat-resisting properties of XLPE insulation materials. Polar functional groups such as carbonyl, hydroxyl, ether and ester group of BHEA would bring in deep traps to capture the charges from the electrodes injecting and the ionization of impurities, and scatter the charge carriers in the transport process, which could raise the potential charge injection barrier and reduce the charge carrier number and mobility to suppress space charge accumulation and reduce the conduction current density of XLPE modified with BHEA.

## Figures and Tables

**Table 1:** Gel content, thermal elongation, mechanical properties, melting temperature and degree of crystallinity of pristine XLPE and XLPE modified with BHEA.

Specimen	XLPE	XLPE/BHEA-0.3	XLPE/BHEA-1.0	XLPE/BHEA-3.0
Gel content	85.2%	89.6%	88.3%	91.1%
Thermal elongation	75%	60%	68%	52%
Tensile strength	26.17	26.94	24.52	24.95
Elongation at break	512.99	502.78	487.85	480.15
Elasticity modulus	195.09	201.96	185.90	211.13
Melting temperature	102.9	103.3	102.5	103.9
Degree of crystallinity	41.07%	40.61%	40.07%	40.06%

## Supplementary Materials

The following are available online at https://www.mdpi.com/2073-4360/11/10/1624/s1, Figure S1: DSC melting thermograms of pristine XLPE and XLPE modified with BHEA, Figure S2: Stress–strain curves of pristine XLPE and XLPE modified with BHEA.
