# Supplementary material for: Improved Direct Current Electrical Properties of Crosslinked Polyethylene Modified with the Polar Group Compound
Source: Polymers (Basel). 2019 Oct 8;11(10):1624. doi: 10.3390/polym11101624 (PMC6835523; doi:10.3390/polym11101624)
Supplement: Supplementary file 1 [file polymers-11-01624-s001.pdf]

polymers

MDPI

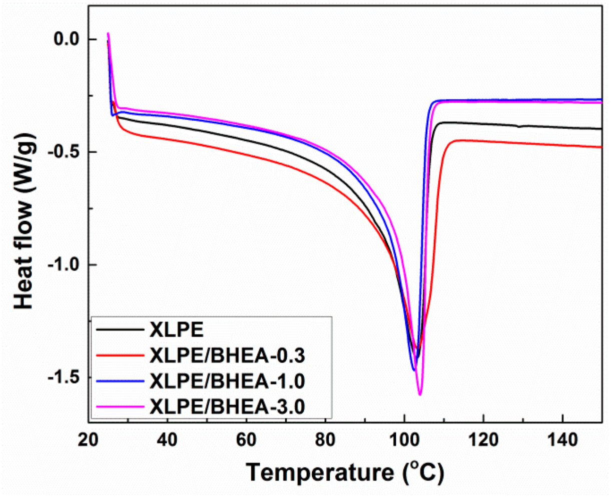

Figure S1. DSC melting thermograms of pristine XLPE and XLPE modified with BHEA.

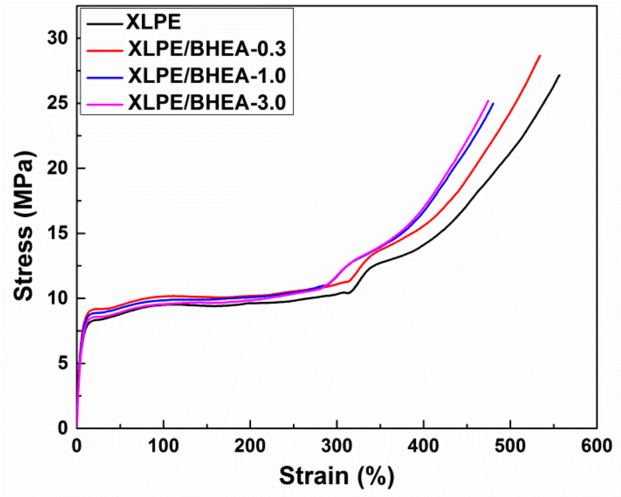

Figure S2. Stress–strain curves of pristine XLPE and XLPE modified with BHEA.
